# Understanding the classics: the unifying concepts of transgressive segregation, inbreeding depression and heterosis and their central relevance for crop breeding

**DOI:** 10.1111/pbi.13481

**Published:** 2020-10-15

**Authors:** Ian J. Mackay, James Cockram, Phil Howell, Wayne Powell

**Affiliations:** ^1^ SRUC (Scotland’s Rural College) Edinburgh UK; ^2^ IMplant Consultancy Chelmsford UK; ^3^ NIAB Cambridge UK

**Keywords:** crop breeding, heterosis, hybrid vigour, transgressive segregation, crop genetics and genomics, quantitative genetics, molecular genetics, genomic selection, heterotic groups

## Abstract

Transgressive segregation and heterosis are the reasons that plant breeding works. Molecular explanations for both phenomena have been suggested and play a contributing role. However, it is often overlooked by molecular genetic researchers that transgressive segregation and heterosis are most simply explained by dispersion of favorable alleles. Therefore, advances in molecular biology will deliver the most impact on plant breeding when integrated with sources of heritable trait variation – and this will be best achieved within a quantitative genetics framework. An example of the power of quantitative approaches is the implementation of genomic selection, which has recently revolutionized animal breeding. Genomic selection is now being applied to both hybrid and inbred crops and is likely to be the major source of improvement in plant breeding practice over the next decade. Breeders’ ability to efficiently apply genomic selection methodologies is due to recent technology advances in genotyping and sequencing. Furthermore, targeted integration of additional molecular data (such as gene expression, gene copy number and methylation status) into genomic prediction models may increase their performance. In this review, we discuss and contextualize a suite of established quantitative genetics themes relating to hybrid vigour, transgressive segregation and their central relevance to plant breeding, with the aim of informing crop researchers outside of the quantitative genetics discipline of their relevance and importance to crop improvement. Better understanding between molecular and quantitative disciplines will increase the potential for further improvements in plant breeding methodologies and so help underpin future food security.

## Introduction

In this review, we comment on recent discoveries in genomics and their relevance to plant breeding. This is motivated by the frequent promotion of such discoveries as causes or mechanisms of heterosis, the phenomenon whereby a filial 1 (F_1_) hybrid outperforms its best parent. We suggest that the search for links between new sources of genomic variation and phenotype should be on heritable trait variation of any kind and not focus on heterosis, and that this will be of greater value in plant breeding. Our belief stems from the commonality between the genetic (rather than mechanistic or genomic) causes of transgressive segregation and heterosis and the fact that heterosis varies between traits within organisms and between organisms for the same trait.

## Transgressive segregation is the reason plant breeding works

**Figure 1:**
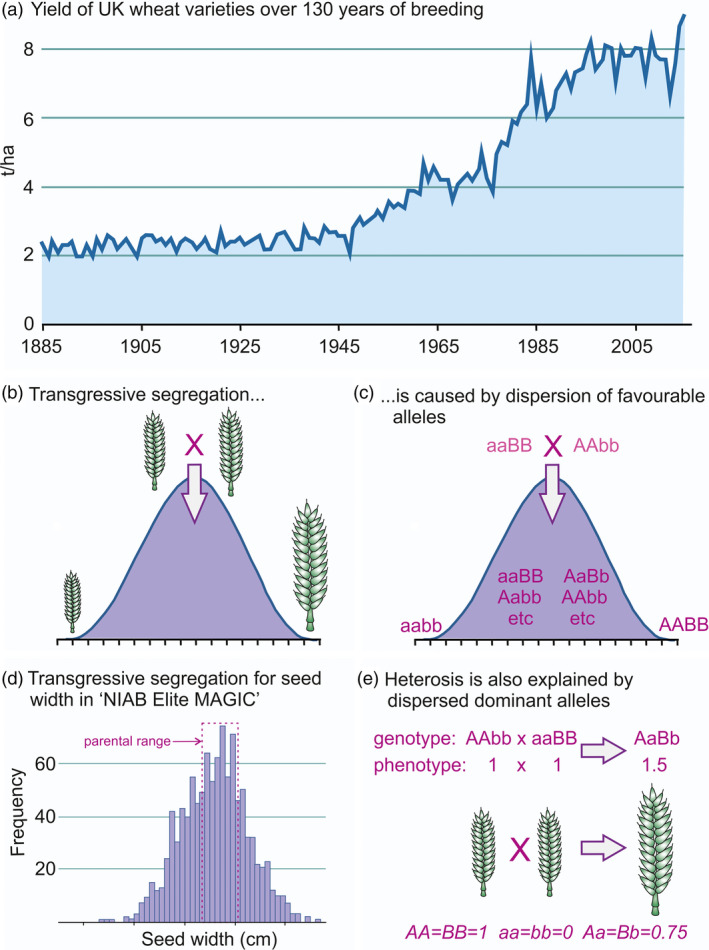
Two sides of the same coin: transgressive segregation and heterosis. (a) The increase in wheat yields in the United Kingdom from 1885 to 2015. Data are taken from the UK Government Web Archive http://www.nationalarchives.gov.uk/webarchive/. (b) Schematic illustrating transgressive segregation in a cross between two elite lines: segregation among progeny (individuals or lines), some of which fall outside the range of trait value for the two parents. (c) Schematic illustrating a simple model for transgressive segregation. The letters a, A, b and B indicate alleles at two loci. Upper case letters increase the value of the trait, lower case letters decrease it. If the increasing alleles are dispersed among the parents of a cross, then segregating progeny can be found which have a higher or lower trait value than the parents. (d) Transgressive segregation for an exemplar trait, seed width, in the ‘NIAB Elite MAGIC wheat’ population (n founders = 8, n progeny = 643, Mackay *et al.,* 2014). Founder trait values are indicated: A = Alchemy, B = Brompton, C = Claire, H = Hereward, Ri = Rialto, Ro = Robigus, S = Soissons, X = Xi19). (e) A simple model for heterosis. The letters a, A, b and B indicate alleles at two loci. Upper case letters are partially dominant. The contribution of each single locus genotype to the trait is AA = BB = 1, Aa = Bb = 0.75, aa = bb = 0. The increasing alleles are dispersed among the parents of a cross. The F_1_ therefore has a higher value than either parent.

Scientific plant breeding is a success. Figure 1a shows the increase in wheat yields in the United Kingdom (UK) from 1885 to 2015, which is mainly the result of breeding (Silvey, 1986; Mackay *et al*., 2011). Indeed, recent work has shown that the genetic improvement for yield over the last 50 years in European wheat has resulted in enhanced cultivar performance under both high‐input and reduced‐input agricultural environments (Voss‐Fels *et al*., 2019). Similar genetic gains for yield are found in other crops (Brisson *et al*., 2010; Laidig *et al*., 2014).

For most naturally self‐pollinating crops (e.g. barley, wheat, oat, soybean, flax), varieties are inbred lines. Breeding occurs through crossing parents, themselves often cultivated varieties, and selecting improved recombinant progeny. If no progeny (or descendants) was ever found which were better than their parents (or ancestors), plant breeding would not work. This property of progeny falling outside the range of the parents is called ‘transgressive segregation’ (Figure 1b). Not all crosses display it, and only a small proportion of progeny in any particular cross may be transgressive, but it occurs frequently enough that plant breeding works as a matter of routine.

A very simple model of gene action can account for transgressive segregation (Figure 1c). Here, genetic variability is determined by multiple genes which contribute additively to the phenotype. In any cross, one parent is fixed for increasing alleles at a proportion of the genes or genetic loci, and the other parent is similarly fixed for increasing alleles at the remainder. Improved progeny lines can then be selected which will be fixed for a greater number of increasing alleles than the better parent. Transgressive segregation for an exemplar trait, seed width, is shown for wheat in Figure 1d: progeny trait values are observed both above and below the parental extremes. When one parent is fixed for all increasing alleles, transgressive segregation is not possible.

This model is of course overly simplistic; it does not take into account interactions between genes (epistasis), genetic linkage, unequal gene effects or the potential for epi‐genetic effects. However, its strength lies in its simplicity, and in many cases fits the observed patterns of genetic segregation and genetic improvement. Genetic analyses invariably find multiple quantitative trait loci (QTL) to be dispersed between parents, even if those parents have contrasting extreme phenotypes. For example, in the Illinois long‐term selection experiment in maize, ~50 QTL for oil content have been identified (Laurie *et al*., 2004). For a fifth of these, the *decreasing* allele was fixed or at higher frequency in the high oil content selection line: selection is not always perfect in fixing favorable alleles, especially for polygenic traits.

With the addition of dominance, this simple model can also explain heterosis. For the purposes of illustrating this point, Figure 1e presents an example with only two loci. Provided that favorable alleles are dispersed between the two parents, and that there is some directional dominance, the performance of the F_1_ will inevitably exceed the best parent. This does not require overdominance (in which the heterozygous genotype at some or all loci outperforms either of the two homozygotes), nor that the increasing allele is dominant at all loci – although it does require that, on balance, the direction of dominance is in the increasing direction. Evidence for directional dominance is easy to find; it is the presence of inbreeding depression (Falconer and Mackay, 1995).

Heterosis and inbreeding depression are, to a considerable extent, opposite sides of the same coin. Different traits in the same species and the same trait in different species show different degrees of inbreeding depression, mirrored by the frequency with which heterosis is found. Examples are yield and quality in virtually any crop: quality generally shows very little or no inbreeding depression, even though the nature of quality is very different between crops. Yield often shows inbreeding depression, but contrasting cases can be found between species even when the physiology of the trait must be very similar. For example, the inbreeding cereals barley and wheat do not show substantial inbreeding depression in grain yield (Longin *et al*., 2012), but their close relative rye (a naturally outcrossing crop) does (Geiger and Miedaner, 1999). Once more, this model is a simplification and complications arise: for example some overdominance may occur and again there may be epistasis, but the model often fits experimental data well (Kaeppler, 2011; Kaeppler, 2012). In crosses with modest heterosis, the inherent expectation is that inbred lines can be selected which outperform the F_1_, and this has been demonstrated in practice (Bradshaw John, 2016; Kearsey and Pooni, 1998).

There are arguments from population and evolutionary genetics, as well as from biochemistry, as to why dominance should be directional [explored, for example, in (Bourguet, 1999; Cornish‐Bowden and Nanjundiah, 2006)]. These are important but not relevant to the themes of this article. The critical points we wish to make at this stage are:

1. Without transgressive segregation, plant breeding would not work.
2. Plant breeding does work; therefore, there is transgressive segregation.
3. Transgressive segregation results from the dispersion of favorable alleles between parents.
4. With directional dominance, heterosis is likely to occur.

These points are of course not original (e.g. Bingham *et al*., 1998) and are found in standard textbooks on quantitative genetics (e.g. Falconer and Mackay, 1995) and plant breeding (e.g. Bradshaw John, 2016). However, they are often overlooked or discounted when researchers in molecular genetics and genomics apply their discoveries to plant breeding. This is notably so in the desire to ‘explain’ heterosis.

## Explanations of heterosis

That F_1_s can yield more than either of their parents have at times been raised to almost mystical status: ‘the mystery of heterosis’, ‘the mysteries of hybrid vigour’, ‘…heterosis remains enigmatic’ and similar statements are easily found. This has been largely fuelled by the huge hybrid advantage in some species. For example, heterosis in hybrid maize relevant to parental lines is >100% (Zanoni and Dudley, 1989) and the historical difficulty in predicting hybrid yield and heterosis (Riedelsheimer *et al*., 2012; Smith, 1986). Although these effects are still largely explained by dispersed dominant loci, many alternative mechanisms to explain or predict heterosis have been proposed. Examples are listed in Table 1, ranging from mitochondrial complementation to circadian clock gene expression. Others can be found in (Feng *et al*., 2015; Kaeppler, 2011; Kearsey and Pooni, 1998; Reif *et al*., 2005).

**Table 1:** Examples of proposed functional mechanisms for heterosis

Mechanism or predictor	Reference
Mitochrondrial complementation	Sarkissian and Srivastava (1967)
Metabolic balance	Hageman *et al*. (1967)
Chloroplast complementation	Srivastava (1981)
Phytohormones, giberellic acid	Rood *et al*. (1988)
DNA methylation	Tsaftaris (1995)
Association transcriptomics	Stokes *et al*. (2010)
Cryptic variation in gene expression	Rosas *et al*. (2010)
Energy‐use efficiency, cell cycle time	Goff (2011)
siRNA	Shivaprasad *et al*. (2012)
sRNA	Barber *et al*. (2012)
Florigen pathway	Jiang *et al*. (2013)
Circadian clock‐mediated stress responses	Miller *et al*. (2015)
Circadian clock gene expression	Shen *et al*. (2015)

Historically, much emphasis has been placed on single locus overdominance as a cause for heterosis. First proposed in 1908 (East, 1908; Shull, 1908), it does indeed seem a mystery: why should the heterozygous class be better than the homozygotes? However, indications that the heterozygote is routinely better than the homozygotes at multiple loci in heterotic crosses are not strong, and evidence has been accumulating against this as a general explanation for at least fifty years (Crow, 1998; Crow, 2000; Kaeppler, 2012; McMullen *et al*., 2009). There are exceptions, of course. For example, overdominance at the *SINGLE FLOWER TRUSS* (*SFT*) locus in tomato contributes substantially to yield heterosis via changes in plant architecture (Krieger *et al*., 2010). On further investigation, examples of single locus overdominance often turn out to result from tightly linked dispersed dominant genes – termed pseudo‐overdominance (Jones, 1917). With increasing understanding of patterns of recombination within the genome, the dispersion of favorable dominant alleles in regions with limited recombination has been found to be common (Mace and Jordan, 2011; McMullen *et al*., 2009). Not least, recombinant inbred lines have been recovered from crosses which exceed the performance of the heterotic F_1_ (Bingham *et al*., 1998; Bradshaw and Wilson, 1993; Jinks and Frankel, 1983), which would not be possible if overdominance was the major source of hybrid vigour in those crosses.

That said, any class of genomic or epi‐genetic variant can make a contribution to heritable variation for economically important traits in domesticated crop species. All the examples listed in Table 1 likely make a contribution, but none will be exclusive. To make a genuine case for a new contributor of genetic variation to any trait is not trivial and is not routinely made. Simply presenting an example of heterosis or of increased genetic variation in a single cross is insufficient, not least because such claims often use inter‐specific crosses and sometimes lack trait data. Finding correlations with heterosis levels over multiple hybrids is inadequate to prove a causal relationship. Just as association mapping shows that spurious patterns of marker–trait association arising from population substructure, rather than from the close linkage of a QTL to the marker, are commonplace (Mackay and Powell, 2007; Yu *et al*., 2006) so any component of genomic or epi‐genetic variation is potentially subject to similar effects. Equivalently, any distinct class of genetic variations, epi‐alleles for example, is likely to be interspersed and in linkage disequilibrium with other classes of variants. The prediction of heterosis, or even additive variation, from epi‐alleles alone, can arise from simple tagging of linked QTL in the same way as single nucleotide polymorphisms (SNPs) tag QTL without being functional polymorphisms.

It has been proposed that structural variants, in particular presence–absence variation (PAV), have greater phenotypic effects than nucleotide variation and can occur at surprisingly high frequencies. For example, (Lai *et al*., 2010) identified 296 genes which were present in the maize reference line B73, but missing from one or more of six elite inbred lines; similarly 157 genes present among these six were missing from B73. Allopolyploids are expected to be more resilient to gene loss and in a recent pangenome study, (Montenegro *et al*., 2017) found PAV for 36% of genes among 18 hexaploid wheat cultivars. The complete absence of a gene seems more likely, a priori, to have a strong phenotypic effect. Although there is no expectation for PAVs to always show directional dominance for increasing expression, dominance in the direction of increasing metabolic flux can be an emergent property for genes affecting metabolic pathways (Kacser and Burns, 1981; Vasseur *et al*., 2019; Wright, 1934). Dispersion of PAVs between the parents of a cross could therefore contribute relatively more to heterosis than dispersion of other classes of variants. However, while the direction of dominance at PAVs may generally be for increasing activity, this is not necessarily the case at the trait level. For heterosis, not only is dominance required, but there must be an excess of loci showing dominance in the same direction. The detection of ambi‐directional dominance is complex but is strongly suggested by the absence of a mean effect of dominance in the presence of variation for dominance. Kearsey *et al*. (2003) studied genetic variation for 22 traits in an Arabidopsis cross and detected an average effect of dominance for eight of these but significant dominance variation for 20. Not all traits show heterosis, and for these any dominance at PAV loci could act in an increasing or decreasing direction. It is easy to imagine cases where a recessive loss of function could act to increase rather than decrease even yield: loss of resistance to disease for example to overcome ‘the cost of resistance’ (Bergelson and Purrington, 1996; Nelson *et al*., 2018). PAV, along with other structural variants such as copy number variation, is important classes of variants with individual effects expected to be greater than for most SNPs at the same locus. It is important to study their effects on trait variation in general and not to focus on heterosis.

Ultimately, if an important component of genetic variation for a specific trait can be accounted for by a molecular process, and if that process can be scored relatively cheaply, robustly and with high throughput, it will be incorporated into practical breeding programmes. However, without evidence that this is the case, practical application may remain challenging.

## Heterotic groups and patterns

A heterotic group consists of lines or individuals which tend to show greater levels of heterosis when crossed outside their group, rather than within it. A heterotic pattern is a pair of groups, such that crosses between groups tend to produce high performing hybrids compared to crosses within groups (Melchinger *et al*., 1998). Heterotic groups and patterns are most simply explained by dispersed dominant loci, though here the dispersion is between groups. A population genetics phenomenon called the Wahlund principle describes the behaviour of partially or completely isolated subpopulations which are then amalgamated (Wahlund, 1928). It shows that if there is divergence in allele frequencies between subpopulations, then there will be greater heterozygosity in crosses between subpopulations than in crosses within subpopulations (e.g. Crow and Kimura, 1970).

Divergence in allele frequency between subpopulations can occur as a result of founder effects, selection or drift. Over multiple loci, if the divergence between subpopulations is agnostic with regards to the frequency of the increasing alleles, dispersion of favorable alleles will result and the best hybrids are more likely to come from between population crosses. This can happen even with selection for the trait, provided the efficiency and direction of selection are similar within subpopulations. The identification of heterotic patterns is therefore a search for subpopulations with diverged allele frequencies and dispersed favorable alleles. This may happen unconsciously: if two breeding programmes, or populations, are kept in isolation in similar environments, chance effects are likely to determine divergence in allele frequencies. However, if two populations are kept in isolation in different environments, or selected for different trait profiles, then populations will still diverge in allele frequencies but now the allelic effects are likely to be associated within subpopulations. As a result, although there will still be greater heterozygosity between populations, the best hybrids may come from crosses within one or other of the subgroups. Finally, if no subgroups exist, they can be created anew by selection and isolation. Table 2 gives some examples of the origins of heterotic groups in maize, rice and rye. In maize breeding, reciprocal recurrent selection is the favored breeding method: new lines are selected from crosses within populations, but selection is made on the performance of inter‐population crosses. This can rapidly result in increased heterosis, divergence between groups and a reduction in the predictability of hybrid performance from the mid‐parental scores. Again, this is expected from the very simple genetic model we have illustrated.

**Table 2:** The origin of commonly used heterotic groups in three crop species

Crop	Origin	Reference
Maize, Europe	Differences between the dent (USA) and flint (EU) heterotic groups pre‐existed	Reif *et al*. (2005)
Maize, USA	Heterotic groups developed during hybrid breeding	Cooper *et al*. (2014b)
Rye, Germany	Petkus and Carsten heterotic groups were identified by a systematic search	Geiger and Miedaner (2009)
Rice, China	Indica I (China) and Indica II (IRRI) groups originated from independent breeding efforts	Xie *et al*. (2015)

## Genomic selection: ‘the quantitative geneticists' revenge’

Quantitative genetics is an integrative science. Applied to plant and animal breeding, the breeders’ equation (Lush, 1937) can predict the effect on rate of response to selection from the introduction of new technologies into extant breeding programmes. In this guise, it has been applied in animal breeding, resulting in more rapid uptake of technologies such as artificial insemination and multiple ovule and embryo transfer (MOET) (Meuwissen, 1998; Raadsma and Tammen, 2005; Visscher *et al*., 2000). The most recent development integrated into animal and plant breeding programmes is the exploitation of cheap, high density, genetic markers into prediction equations for traits through the process of genomic selection (Jannink *et al*., 2010; Meuwissen *et al*., 2001). This has revolutionized dairy cattle breeding over the last ten years and is in the process of revolutionizing the breeding of other animal species (Hayes *et al*., 2013). Routine use of genomic selection in plant breeding has been lower but it is also now increasingly applied in commercial plant breeding programmes (e.g. maize Cooper *et al*., 2014a)) and also used to predict which parents to cross to make the best hybrids (Zhao *et al*., 2015). In the short to medium term, improvements in plant breeding are more likely to come from the application of genomic selection than from any other technology (Mackay *et al*., 2019).

The phrase ‘the quantitative geneticists' revenge’ was applied in jest to genomic selection by Alan Archibald of the Roslin Institute to illustrate the discipline's escape from the conventional restrictions of QTL mapping, molecular biology and physiology to simply predict trait performance directly from DNA sequence. In this respect, its aims are modest: it makes no attempt to advance the understanding of the biology of important quantitative traits but has been proven to make predictions which work in practice. Genomic selection methods have also been used to test the integration of transcriptomic and/or metabolomic data into trait prediction (Riedelsheimer *et al*., 2012; Schrag *et al*., 2018; Ward *et al*., 2015) applicable to both inbred and hybrid cultivars, and could also be used to integrate any other novel approach, such as heritable variation in methylation patterns (Boulesteix *et al*., 2017). However, it must first be demonstrated that these new discoveries are important contributors to variation in breeder‐relevant traits.

The statistical methods used in genomic selection generally have little bearing on the accuracy of trait prediction and research in crops is increasingly focused on to how best to implement genomic selection in breeding programmes (Mackay *et al*., 2019). In this respect, rather than using methods to predict the trait values of selection candidates directly, extensions to methods have been made to predict the merit of crosses: either the performance of the hybrid, or the distribution of lines descended from the cross. An example is to predict the proportion of lines which exceed a specified target: the ‘usefulness’ of the cross (Lehermeier *et al*., 2017). If the target is the real or predicted trait value of a parent, this amounts to predicting the probability of transgressive segregation. Combining such approaches with the methods to integrate ‘omics variation such as that described above can provide a survey of the relative contribution of all sources of genomic and ‘omic variation to traits. For example, in a comprehensive study of a maize diallel, (Yang *et al*., 2017) established that alleles predicted to have deleterious effects on fitness tended to be incompletely dominant and contributed substantially to trait variation and heterosis. Taking this into account improved trait prediction accuracy.

## Heterosis at other ploidy levels

Most theoretical and practical discussion of heterosis in crops considers diploids and allopolyploids (polyploids with chromosomes derived from two or more diverged taxa, e.g. cotton, peanut and canola – also known as oilseed rape). The latter behave in meiosis as diploids. There may be an expectation of finding more epistasis as a cause of heterosis, and in allopolyploid wheat, this was thought to be the case. However, in allohexaploid wheat, (Santantonio *et al*., 2019) found although homoeologous interactions explain a portion of the non‐additive genetic signal, the contribution is less than other sources of epistasis.

**Figure 2:**
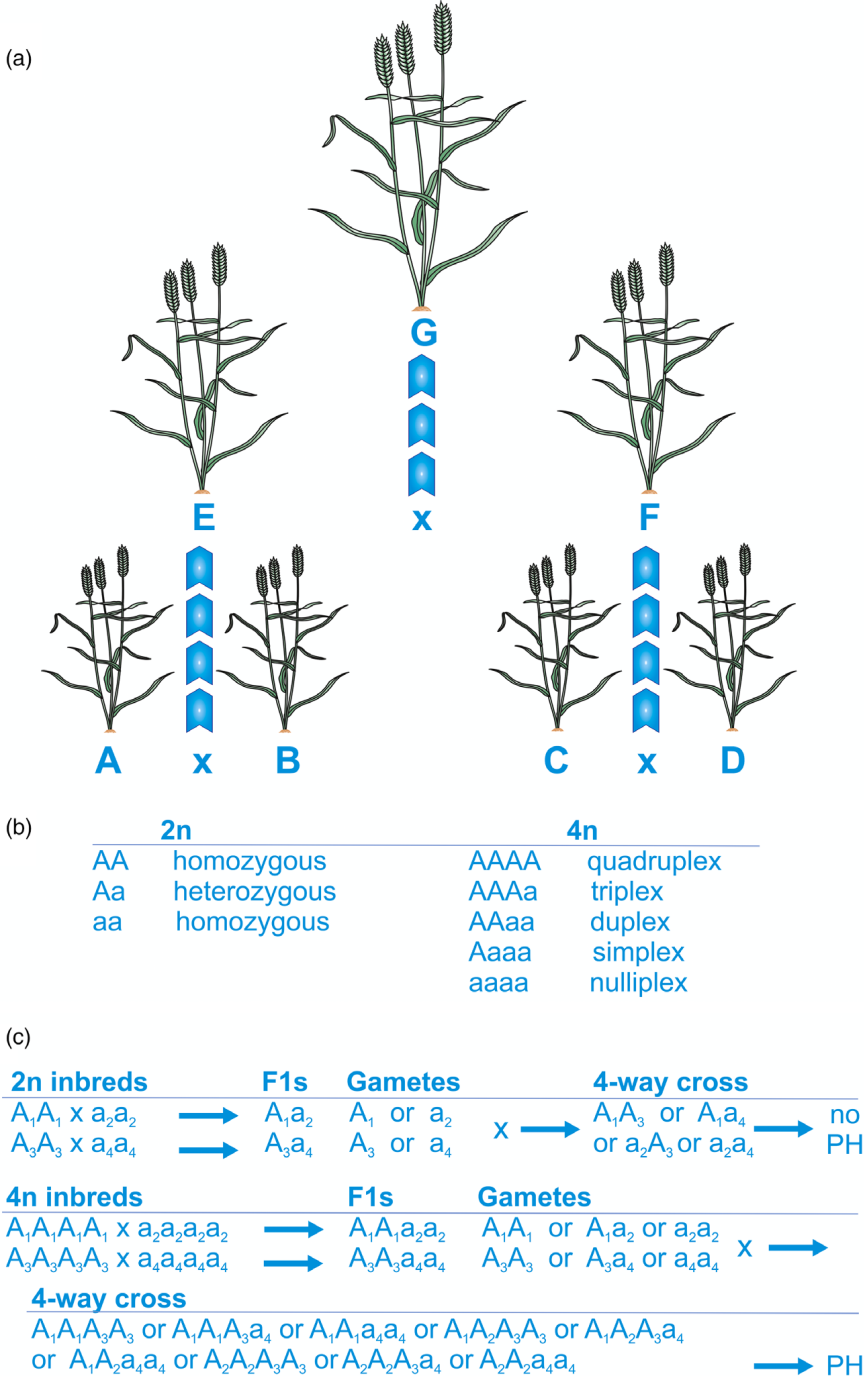
Comparison of heterosis in diploid (2n) and tetraploid (4n) inbred crops, and an explanation for progressive heterosis based on dispersion of dominant alleles. (a) Illustration of progressive heterosis where four inbred tetraploid lines (A, B, C, D) are crossed in pairs (AxB and CxD), and the resulting hybrids (E and F) subsequently crossed with each other to create double‐cross hybrids (G) that show greater heterosis on average than either of the single‐cross hybrids. (b) Possible genotypes at a single locus with two alleles in a diploid and tetraploid. (c) Illustration of progressive heterosis (PH) in a diploid and a tetraploid, based on four alleles, A1 A2 a3 a4 where Ax is partially dominant to ay.

The study of genetic interactions in autopolyploids (polyploids with chromosomes derived from a single taxon, e.g. potato) is more complex (Figure 2a). When restricted to bi‐allelic models, there are three possible heterozygous classes (simplex, duplex and triplex; Figure 2b), with the addition of multiple alleles adding complexity. Autopolyploids generally show very strong inbreeding depression. However, as many domesticated autopolyploids are clonally propagated (e.g. sugar cane, banana, grape), their breeding has not focused on the development of hybrid varieties. Consideration of causes of heterosis in autopolyploids is confounded with considerations about their evolution and the differences in the expected rates of progress that can be made through breeding at the diploid level (fast) and in polyploids (slow). In the past, the success of autopolyploids has been attributed to their greater heterozygosity and multiple‐alleleism. Just as for diploids, this can be explained by virtually any genetic model.

Potatoes, clonal autotetraploids, are a case in point. There are interest and research in developing true seed F_1_ hybrid diploid potatoes, with some reported success (Stokstad, 2019). Part of the reason for the development of these F_1_s is non‐genetic: true seed can be transported and stored more easily than seed potato tubers, and this is particularly important in the developing world, where their uptake is more advanced. However, (Muthoni *et al*., 2019) conclude that there is little experimental evidence to support any superiority of diploids over tetraploids and that the theory that heterosis for yield in potato may be achieved by maximizing heterozygosity remains unchallenged. This is supported by the phenomenon of ‘progressive heterosis’ [Washburn and Birchler, 2014; Figure 2a] in which progeny from a 4‐way cross shows heterosis over its two parental F_1_s, which in turn shows heterosis over their parents. This is observed in potatoes and other autotetraploid species. Washburn *et al*. (2019) studied progressive heterosis in maize, crossing pairs of diploid inbreds to make two F_1_s which were in turn used as parents for a 4‐way cross, but in addition repeating this crossing scheme at the tetraploid level using tetraploid versions of the four diploid inbreds. In extensive testing, they found progressive heterosis at the tetraploid level for several traits (48% for above ground dry weight) but none at the diploid level.

Although conditions for an autotetraploid F_1_ between two inbred lines to show heterosis under a simple quantitative genetic model are equivalent to those at the diploid level (net directional dominance of duplex genotypes and dispersion of favorable alleles between the parents), conditions for progressive heterosis are complex (Figure 2c): the four‐way cross is segregating and the average heterosis of the genotypes depends on the dominance relationships of the simplex, duplex and triplex genotypes, the complexities of autotetraploid inheritance, and the number of alleles segregating at each locus. Heterosis in autopolyploids warrants further experimental study.

## Epistasis and inbreeding species

There is increasing interest in developing hybrids in inbreeding species, most notably in the cereal crops wheat and barley, and in partially selfing species such as canola. Although the levels of heterosis are lower (for example 10% in wheat (Jiang *et al*., 2017) compared to over 100% in maize (Zanoni and Dudley, 1989), as expected since deleterious recessive alleles are exposed to selection in homozygotes at a higher frequency), the gains are economically important as long as cost‐efficient F_1_ seed production methodologies can be developed. In rice, >50% of the crop in China are hybrids, facilitated by the recent introduction of practical systems for F_1_ seed production. Empirically, there is evidence that heterosis in inbreeding crops tends to result from epistasis as much as from dominance (Jiang *et al*., 2017). Charlesworth and Willis (2009) give a cartoon example whereby seed size and seed number are inherited additively but when multiplied to produce yield, can show heterosis which would be attributed to additive × additive interactions among loci determining yield. The consequences of such gene action for breeding programmes remain largely unexplored. To illustrate this, with the simple example of (Charlesworth and Willis, 2009), the same F_1_ can be produced by different pairs of inbreds to give either positive or negative heterosis.

Rice is a successful example of the development of hybrid breeding for an inbreeding species. Natural outcrossing rates are very low (Messeguer *et al*., 2001) but hybrids now account for over half of rice cultivation 50% of the total rice area in China, India and Indonesia (Chen, 2010). Heterosis in rice has been found to be associated with incomplete dominance (Huang *et al*., 2015; Huang *et al*., 2016; Xiao *et al*., 1995), overdominance (Li *et al*., 2001) and epistasis (Yu *et al*., 1997). It seems likely that all are involved with relative contributions depending on population and study methods.

## Exceptions and future developments

We regard dispersion of favorable alleles between parents as the underlying cause of most heterosis and of transgressive segregation, and that all sources of genomic variation are likely to affect heterotic and non‐heterotic variation; it is improbable there is a universal explanation for heterosis. New discoveries in genomics should be judged against this baseline. However, there are also interesting exceptions, areas for further study and development, and unknowns. For example, ‘hybrid decay’ has recently been described in maize, whereby the F_1_ between maize and an accession from its ancestral species, teosinte, appears normal looking (for a wide cross of this type), but shows a sickly phenotype when backcrossed to maize. Furthermore, this increases in severity in subsequent backcrosses, rather than being eliminated as the parental genome is recovered (Xue *et al*., 2019). This ‘hybrid decay’ was non‐Mendelian, epi‐genetic and possibly due to the activation and amplification of previously silenced transposable elements in the teosinte genome.

## Conclusions

The two most important phenomena in plant breeding are transgressive segregation and heterosis. Both are most simply explained by dispersion of favorable alleles, which in the case of heterosis must also show directional dominance. Additional factors including epistasis, overdominance and linkage are of varying importance in some instances, but the most basic model of plant breeding works well in practice. Further advances in molecular biology will undoubtedly have an impact on plant breeding, and this will be maximized if their importance is understood and integrated with known sources of heritable trait variation. Accordingly, molecular biology will best deliver impact to breeding by integrating novel discoveries within a quantitative genetics framework. For the major crops, genomic selection is now being applied to both hybrid and inbred crops by commercial companies, and this is likely to be the major source of genetic improvement in polygenic traits such as yield over the next decade.

## Conflict of interest

The authors declare they have no conflict of interest.

## Authors’ contributions

IM conceived the article. JC, PH and WP contributed to the development of the ideas expressed within it. IM wrote the manuscript, with contributions from JC, PH and WP.
